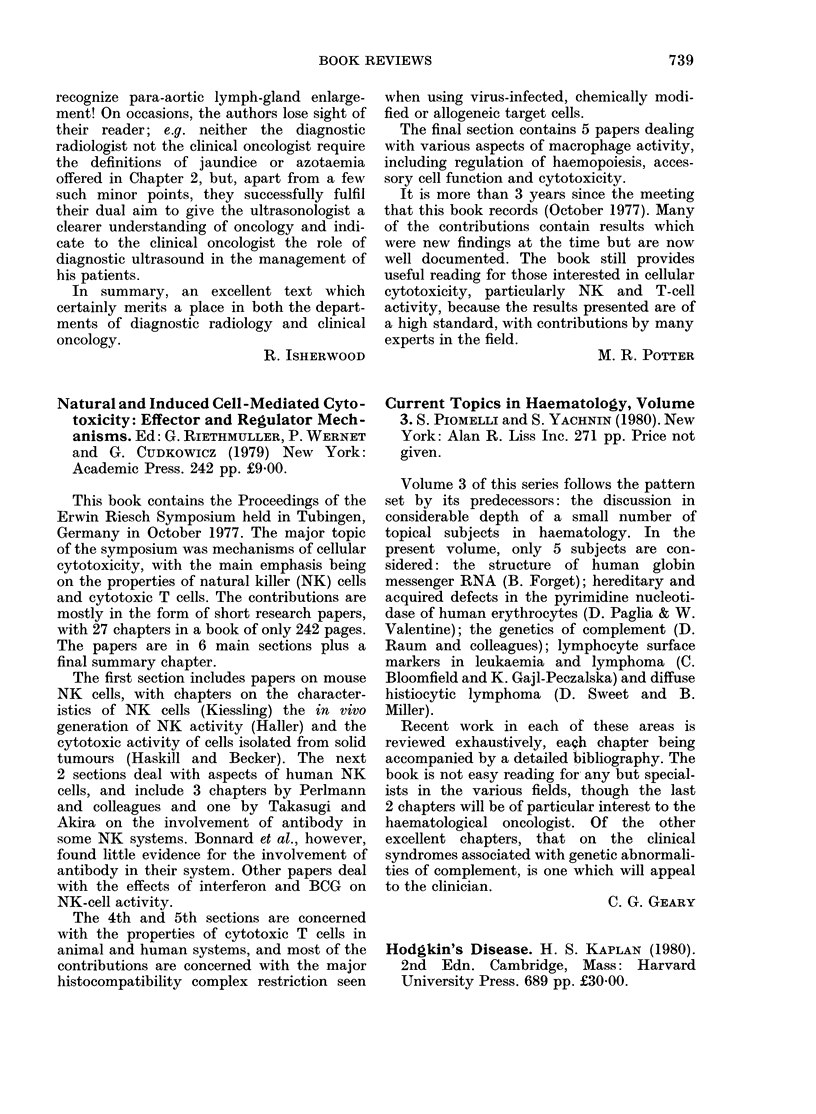# Current Topics in Haematology, Volume 3

**Published:** 1981-05

**Authors:** C. G. Geary


**Current Topics in Haematology, Volume 3.** S. PIOMELLI and S. YACHNIN (1980). New York: Alan R. Liss Inc. 271 pp. Price not given.

Volume 3 of this series follows the pattern set by its predecessors: the discussion in considerable depth of a small number of topical subjects in haematology. In the present volume, only 5 subjects are considered: the structure of human globin messenger RNA (B. Forget); hereditary and acquired defects in the pyrimidine nucleotidase of human erythrocytes (D. Paglia & W. Valentine); the genetics of complement (D. Raum and colleagues); lymphocyte surface markers in leukaemia and lymphoma (C. Bloomfield and K. Gajl-Peczalska) and diffuse histiocytic lymphoma (D. Sweet and B. Miller).

Recent work in each of these areas is reviewed exhaustively, each chapter being accompanied by a detailed bibliography. The book is not easy reading for any but specialists in the various fields, though the last 2 chapters will be of particular interest to the haematological oncologist. Of the other excellent chapters, that on the clinical syndromes associated with genetic abnormalities of complement, is one which will appeal to the clinician.

C. G. GEARY